# Machine Learning Comparison and Combined Model Optimization Based on DCE‐MRI Radiomics for Preoperative Assessment of Lymphovascular Invasion in Breast Cancer: A Multicenter Study

**DOI:** 10.1002/cnr2.70678

**Published:** 2026-09-13

**Authors:** Hongen Li, Qingwen Xiao, Yihui Zeng, Xia Wang, Yan Zhang

**Affiliations:** ^1^ Department of Radiology Guangdong Women and Children Hospital Guangzhou China; ^2^ The First Affiliated Hospital of Jinan University Guangzhou China

**Keywords:** breast cancer, machine learning, magnetic resonance imaging, radiomics, vascular invasion

## Abstract

**Purpose:**

To explore the value of constructing a radiomics combined model based on preoperative dynamic contrast‐enhanced magnetic resonance imaging (DCE‐MRI) for predicting lymphovascular invasion (LVI) in breast cancer.

**Materials and Methods:**

Retrospective data collection was performed from December 2022 to November 2025, involving 912 patients with pathologically confirmed breast cancer who underwent DCE‐MRI examinations at two medical centers. Data from 757 patients at Center 1 (Guangdong Provincial Maternal and Child Health Hospital) were used as the model development set. Through stratified random sampling, this set was divided into a training set (*n* = 529) and an internal validation set (*n* = 228) at a 7:3 ratio. The 155 patients from Center 2 (The First Affiliated Hospital of Jinan University) formed an independent external test set. Three‐dimensional regions of interest (ROIs) were manually delineated on DCE‐MRI images, and 1197 radiomics features were extracted using the PyRadiomics software. Within the training set, key features were selected through univariate analysis (*p* < 0.05) using Pearson correlation analysis (|*r*| < 0.9) and LASSO regression (10‐fold cross‐validation). Key features were evaluated using logistic regression (LR), support vector machine (SVM), K‐nearest neighbors (KNN), random forest (RF), extreme trees (ET), extreme gradient boosting (XGBoost), light gradient boosting machine (LightGBM), gradient boosting machine (GBM), adaptive boosting (AdaBoost), and multilayer perceptron (MLP) to construct radiomics models, identifying the model with the highest predictive performance. Simultaneously, univariate and multivariate logistic regression analyses were employed to identify independent risk factors for breast cancer LVI from clinical and pathological characteristics, and a clinical model was constructed. A combined model was constructed by integrating the independent risk factors for breast cancer LVI with the optimal radiomics model, and a nomogram was developed. The predictive performance and clinical utility of each model for breast cancer LVI were evaluated using receiver operating characteristic (ROC) curves, calibration curves, and decision curve analysis (DCA).

**Results:**

Based on DCE‐MRI images, 18 key features were ultimately selected for constructing the radiomics model. Among these, the ExtraTrees radiomics model demonstrated the highest predictive performance, achieving an area under the curve (AUC) of 0.812 (95% CI: 0.7768–0.8480) on the training set and 0.653 (95% CI: 0.5977–0.7081), with accuracy superior to the other nine machine learning models in the validation set. Independent risk factors for breast cancer LVI included sentinel lymph node status, ER, and TIC, which were used to construct a clinical model. The AUC values for the training set were 0.700 (95% CI: 0.6557–0.7440), 0.752 (95% CI: 0.7101–0.7933), and 0.796 (95% CI: 0.7582–0.8341). The corresponding AUC values on the validation set were 0.642 (95% CI: 0.5695–0.7145), 0.664 (95% CI: 0.5901–0.7374), and 0.704 (95% CI: 0.6339–0.7745). The AUC values for the test set were 0.603 (95% CI: 0.5131–0.6923), 0.692 (95% CI: 0.6097–0.7739), and 0.703 (95% CI: 0.6187–0.7866). Calibration curves demonstrated good agreement between the combined model's predictions and actual observed outcomes. DCA indicated that the model provided greater clinical net benefit across a broad range of threshold probabilities.

**Conclusions:**

The combined model constructed based on DCE‐MRI effectively predicts LVI in breast cancer, demonstrating superior performance compared to standalone radiomics or clinical models. It exhibits excellent calibration and clinical utility, offering promising potential to support preoperative individualized treatment decisions.

Globally, breast cancer has consistently ranked as the leading cause of malignant tumors among women for many consecutive years, posing a significant public health burden [1]. Projections from the International Agency for Research on Cancer (IARC) indicate that the global incidence of new breast cancer cases and mortality burden will continue to rise in the coming decades [2]. Against this backdrop, achieving precise personalized treatment is crucial for improving patient prognosis. Lymphovascular invasion (LVI), also known as vascular invasion, refers to the presence of tumor cells within distinct endothelial spaces (lymphatic vessels or blood vessels) in the peritumoral space of invasive breast cancer, and/or the involvement of peritumoral lymphatic spaces or blood vessels by tumor emboli [3, 4, 5]. Research indicates that LVI, as a significant pathological indicator in breast cancer, has been widely established as an independent predictor of local tumor recurrence, distant metastasis, and poor prognosis [6, 7]. Precise preoperative assessment of LVI status assists clinicians in formulating more tailored surgical plans (e.g., axillary lymph node dissection extent) and adjuvant treatment strategies. However, current confirmation of LVI relies entirely on postoperative pathological examination. Although several imaging‐based and radiomics studies have explored preoperative LVI prediction, these promising noninvasive approaches are not yet sufficiently validated or standardized for routine clinical use. Therefore, there is an urgent clinical need to develop and rigorously validate a robust noninvasive method capable of accurately predicting LVI status prior to surgery.

Dynamic contrast‐enhanced magnetic resonance imaging (DCE‐MRI) has become a core modality for preoperative breast cancer assessment due to its exceptional soft‐tissue resolution and ability to evaluate tumor vascularization characteristics. Radiomics provides a powerful tool for noninvasively decoding tumor heterogeneity by high‐throughput extraction and analysis of quantitative features within images that are invisible to the naked eye [8, 9]. In recent years, researchers have attempted to predict breast cancer LVI using radiomics based on digital breast tomosynthesis (DBT) and other imaging modalities, achieving some progress [10, 11]. However, research on LVI prediction using DCE‐MRI—a more widely applied and information‐rich imaging technique in breast cancer assessment—remains relatively scarce. Existing studies also exhibit several limitations: most adopt single‐center designs, employ a limited variety of machine learning algorithms (e.g.,), logistic regression, support vector machines, and lack of a clinically interpretable combined model. Further exploration is warranted.

Given this, the present study aims to develop and rigorously validate a combined model based on multicenter DCE‐MRI for preoperative prediction of breast cancer LVI. Specifically, we retrospectively collected DCE‐MRI data from 912 breast cancer patients across two independent medical centers. In the LVI prediction study, we first extracted a large number of radiomics features from medical images and performed feature selection through a rigorous multistep process. Subsequently, we systematically constructed and compared 10 distinct machine learning models to identify the optimal radiomics model performing best on an independent validation set. Concurrently, we determined independent clinical risk factors for predicting LVI through multivariate analysis. Finally, we integrated the optimal radiomics model with these clinical risk factors to build a comprehensive predictive model, visualized as a nomogram. This study employed a rigorous multicenter external validation strategy and extensively compared multiple algorithms to ensure the developed preoperative noninvasive prediction tools possess robust generalizability and stable performance. These tools underwent meticulous discrimination and calibration assessments, as well as validation across diverse populations, aiming to provide strong evidence for personalized precision treatment decisions in breast cancer.

## 1 Materials and Methods

### 1.1 Clinical Data

This study is a retrospective, multicenter investigation conducted in accordance with the Declaration of Helsinki. The research protocol was approved by the institutional medical ethics committees of both participating centers (Center 1: Guangdong Maternal and Child Health Hospital, Approval No. 20261015; Center 2: The First Affiliated Hospital of Jinan University, Approval No. KY‐2025‐180), and patients were exempted from providing informed consent.

**FIGURE 1:**
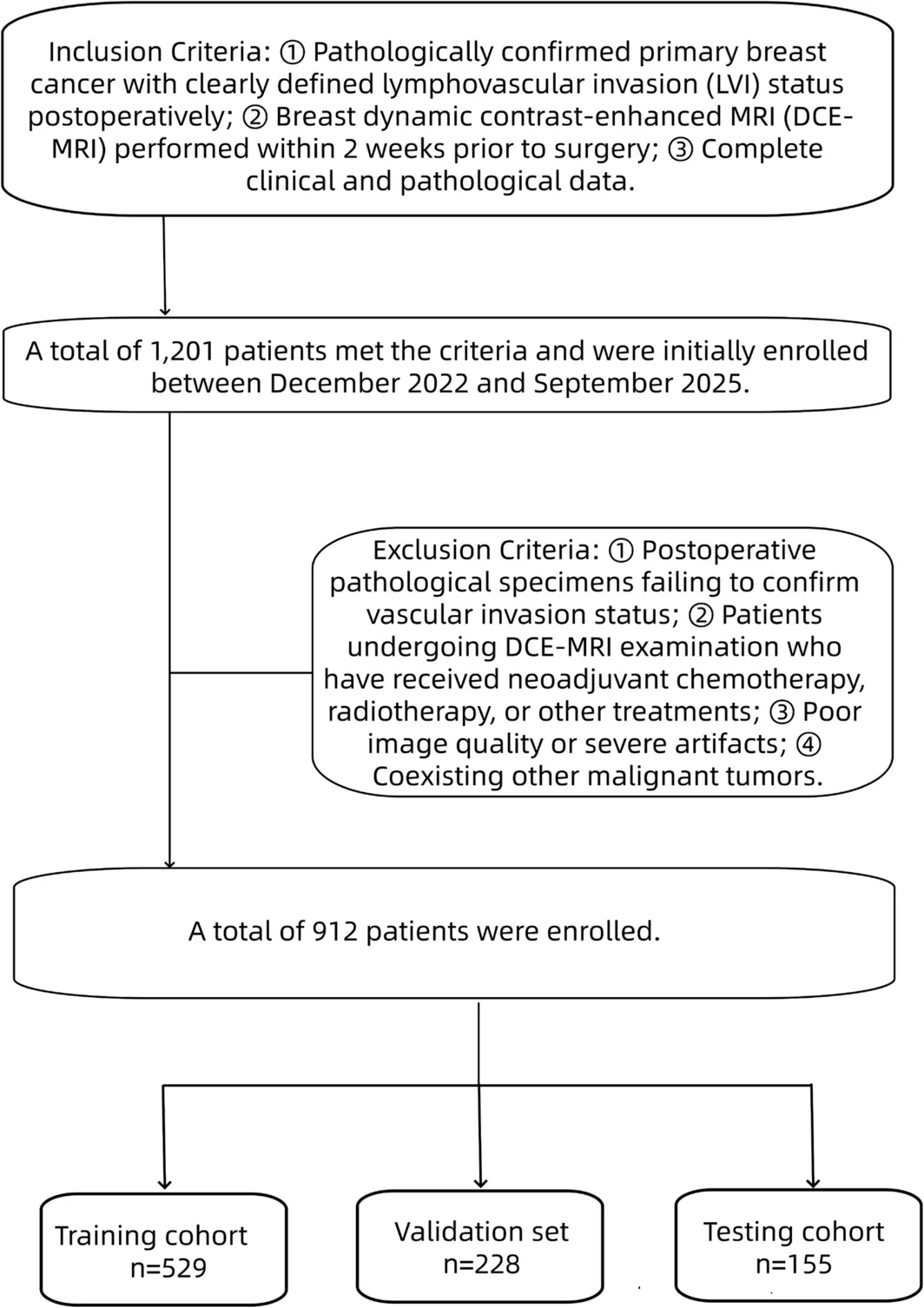
Patient enrollment flowchart.

Between December 2022 and November 2025, breast cancer patients who underwent preoperative breast magnetic resonance imaging (MRI) at two centers were retrospectively screened. *Inclusion criteria:* ① Pathologically confirmed primary breast cancer with defined LVI status; ② Breast dynamic contrast‐enhanced MRI (DCE‐MRI) performed within 2 weeks preoperatively; ③ Complete clinical and pathological data. *Exclusion criteria:* ① Postoperative pathology specimens failing to confirm vascular/lymphatic invasion status; ② Patients undergoing neoadjuvant chemotherapy, radiotherapy, or other treatments during DCE‐MRI examination; ③ Poor image quality or severe artifacts; ④ Concurrent other malignant tumors. Ultimately, 912 female patients were enrolled (enrollment flow diagram shown in Figure 1), ranging in age from 23 to 81 years, with a mean age of (50.7117 ± 10.74) years.

To rigorously evaluate the model's generalization capability, we adopted a multicenter external validation strategy. Data from 757 patients at Center 1 (Guangdong Provincial Maternal and Child Health Hospital) constituted the model development set. Within this development set, we employed stratified random sampling (based on LVI status) to partition the data into a training set (train, *n* = 529) and an internal validation set (val, *n* = 228) at a 7:3 ratio, ensuring consistent LVI distribution across both subsets. Data from 155 patients at Center 2 (The First Affiliated Hospital of Jinan University) served as an independent, unseen external test set (test).

### 1.2 Inspection Method

Scans were performed using a Philips Ingenia 3.0T MRI scanner with a 16‐channel phased array coil specifically designed for breast imaging. Specific scanning sequences and parameters are as follows: ① Transverse T1‐weighted imaging (T1WI): Repetition time (TR) 400–600 ms, Echo time (TE) 8 ms, slice thickness 4 mm, slice spacing 0.4 mm; ② Fat‐suppressed transverse T2‐weighted imaging (T2WI): TR 3000–5000 ms, TE 75 ms, slice thickness 4 mm, slice spacing 0.4 mm; ③ Transverse diffusion‐weighted imaging (DWI) parameters: TR 6550 ms, TE 99 ms, slice thickness 4 mm, slice spacing 0.4 mm, *b*‐values 0 and 800 s/mm^2^; Transverse dynamic contrast‐enhanced MRI (DCE‐MRI) parameters: TR 4.4 ms, TE 2.2 ms, slice thickness 1.6 mm, slice spacing −0.8 mm. Gadoteric acid gadolinium ethylsuccinate injection (0.2 mmol/kg body weight) was administered intravenously at a flow rate of 2.0 mL/s, followed by 15 mL saline flush. Six consecutive contrast‐enhanced phases were acquired. The third phase (arterial phase, approximately 90–120 s post‐injection) was used for subsequent radiomics feature extraction and analysis.

### 1.3 Clinical Pathology and MRI Semantic Feature Evaluation

Two radiologists with over 10 years of experience in breast imaging diagnosis independently evaluated all MRI images without prior knowledge of pathological results. Semantic features assessed included: lesion type (mass‐like vs. non‐mass‐like enhancement), Time‐intensity curve (TIC) pattern (Type I: sustained rise; Type II: plateau; Type III: washout), MRI‐assessed axillary lymph node status (positive vs. negative), and tumor maximum diameter. In cases of disagreement, a third senior radiologist (with > 20 years of experience) arbitrated to reach consensus.

The pathological definition of LVI is: clusters of cancer cells visible within spaces surrounded by flattened endothelial cells under light microscopy, and contiguous with the flattened endothelial cells. Clinical pathological characteristics were collected from the electronic medical record system, including age, menopausal status, family history of breast cancer, histological grade, sentinel lymph node status, and biological markers. Biological markers primarily include estrogen receptor (ER), progesterone receptor (PR), human epidermal growth factor receptor 2 (HER‐2), and the cell proliferation index (Ki‐67). ER and PR assessment criteria: < 1% positive nuclei are negative, ≥ 1% are positive. HER‐2 assessment criteria [12]: Scores of “−” or “+” indicate negative status, while “+++” indicates positive status. Scores of “++” require further fluorescence in situ hybridization (FISH) testing, with gene amplification indicating a positive status and no amplification indicating a negative status. Ki67 criteria: ≥ 14% is high expression; < 14% is low expression. According to the 2013 St. Gallen International Breast Cancer Conference consensus [13], breast cancer molecular subtypes are clearly defined into four categories: Luminal A (ER/PR‐positive, HER2‐negative, low Ki67 expression), Luminal B (further subdivided into HER2‐negative and HER2‐positive subtypes), HER2‐overexpressing (HER2‐positive, ER and PR‐negative), and triple‐negative (ER, PR, and HER2 all negative).

### 1.4 Radiomics Analysis of MR Images

**FIGURE 2:**
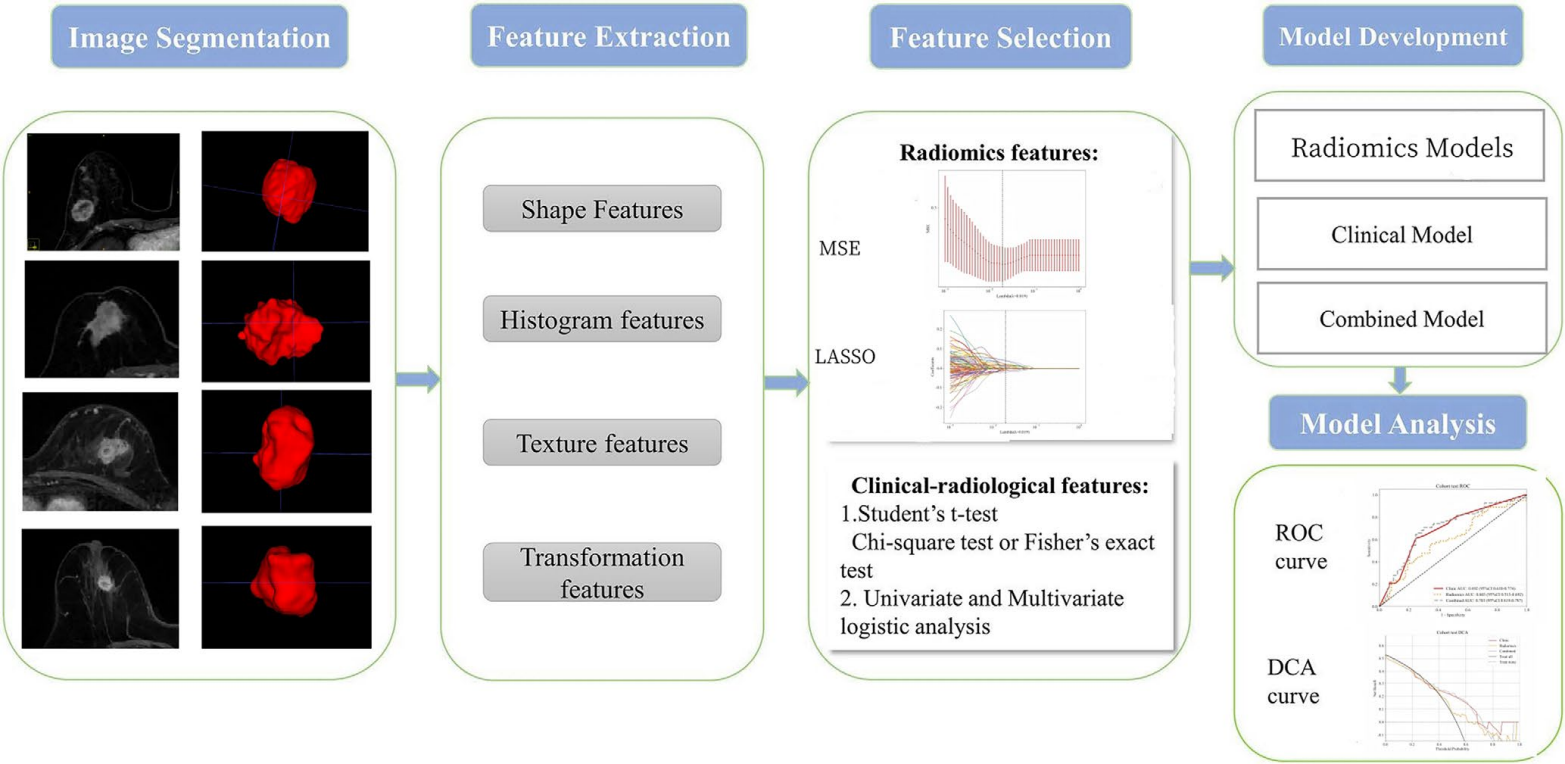
Workflow of this study.

The radiomics analysis workflow includes image segmentation, feature extraction, feature selection, model building, and validation (Figure 2).

#### 1.4.1 Image Segmentation

DCE‐MRI images were imported from the Picture Archiving and Communication System (PACS) into the ITK‐SNAP 3.8 statistical software (http://www.itksnap.org). The third post‐contrast phase (arterial phase, acquired approximately 90–120 s after contrast injection) of the six‐phase DCE‐MRI sequence was selected for radiomics analysis. Non‐subtracted original images were used, and a single‐phase (uniphasic) radiomics analysis was performed. A radiologist with 8 years of experience manually contoured the entire tumor region layer by layer on the selected DCE‐MRI images, generating a three‐dimensional volume of interest (VOI). Subsequently, another physician with over 10 years of experience reviewed the delineations. If significant discrepancies existed between the two delineations, a third senior expert (with > 20 years of experience) made the final determination. All delineations were performed without knowledge of clinical or pathological information.

#### 1.4.2 Feature Extraction and Screening in Radiomics

**FIGURE 3:**
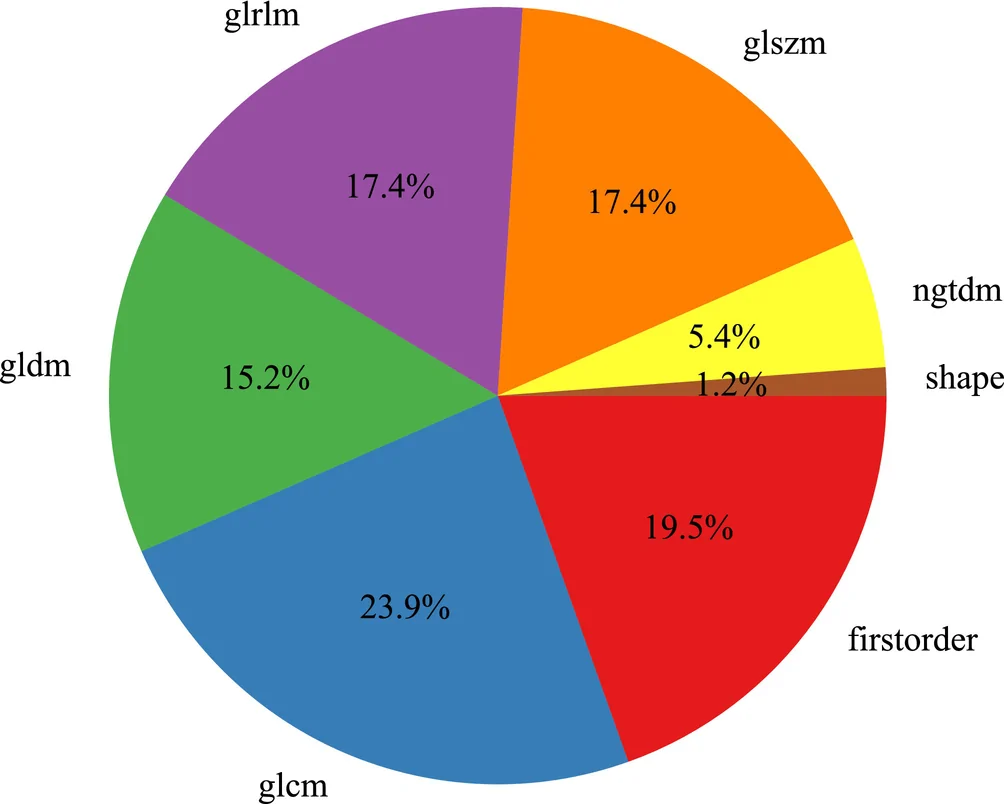
Number and ratio of handcrafted features.

**FIGURE 4:**
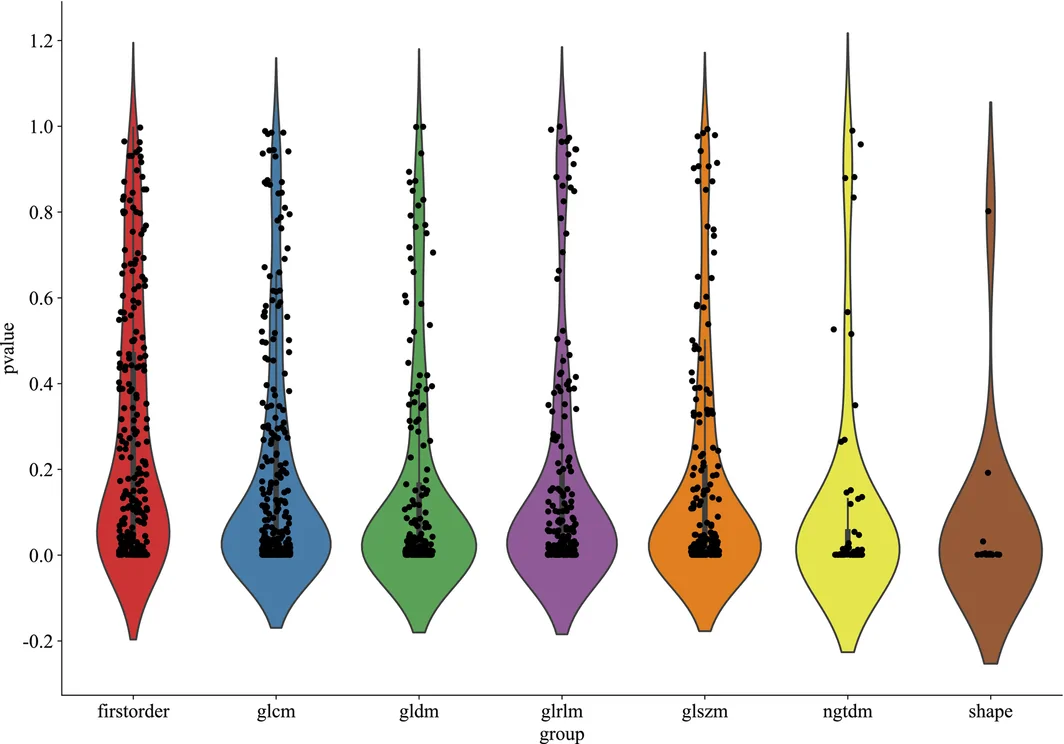
Statistics of radiomic features.

PyRadiomics (version 3.7.12, https://pyradiomics.readthedocs.io) was used to extract radiomics features from the VOI of each patient. Prior to feature extraction, raw images underwent normalization using the Z‐score standardization method to adjust gray values to a normal distribution, enhancing comparability between datasets. Based on each patient's images, 1197 radiomics features were extracted, comprising 234 histogram features, 14 morphological features, 884 texture features, and 65 higher‐order features (see Figures 3 and 4).

**FIGURE 5:**
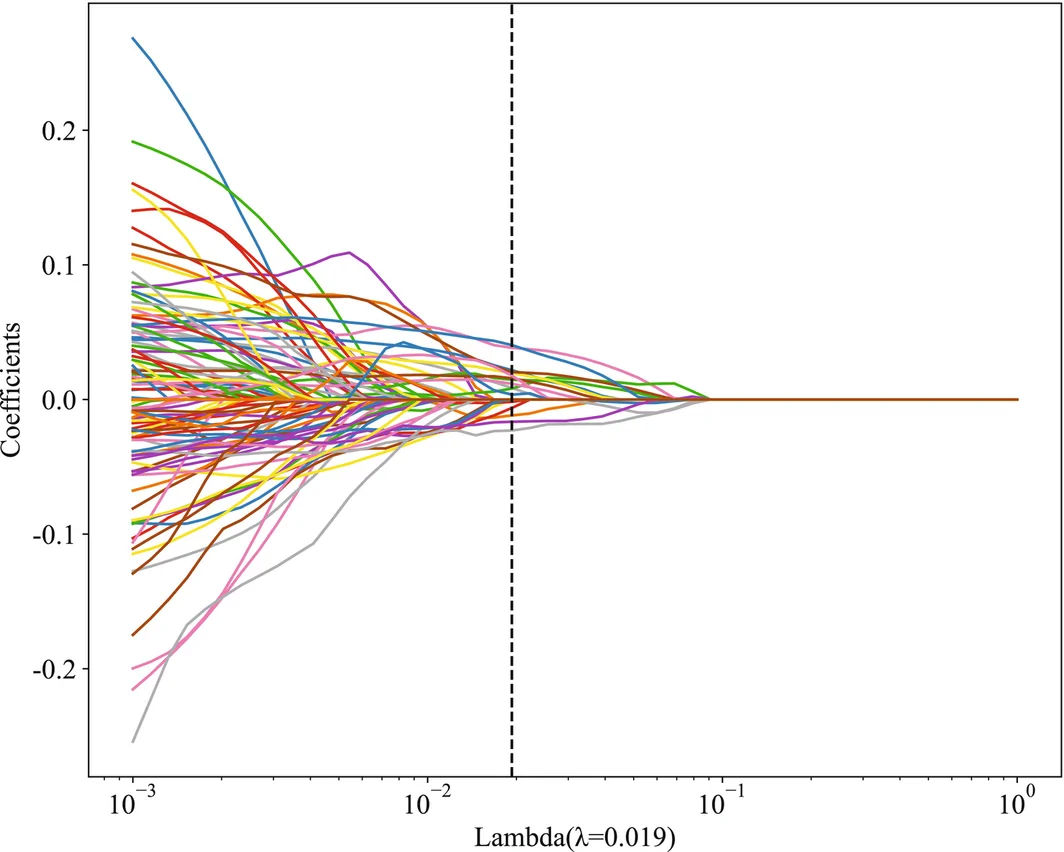
Coefficients from the 10‐fold cross‐validation process.

**FIGURE 6:**
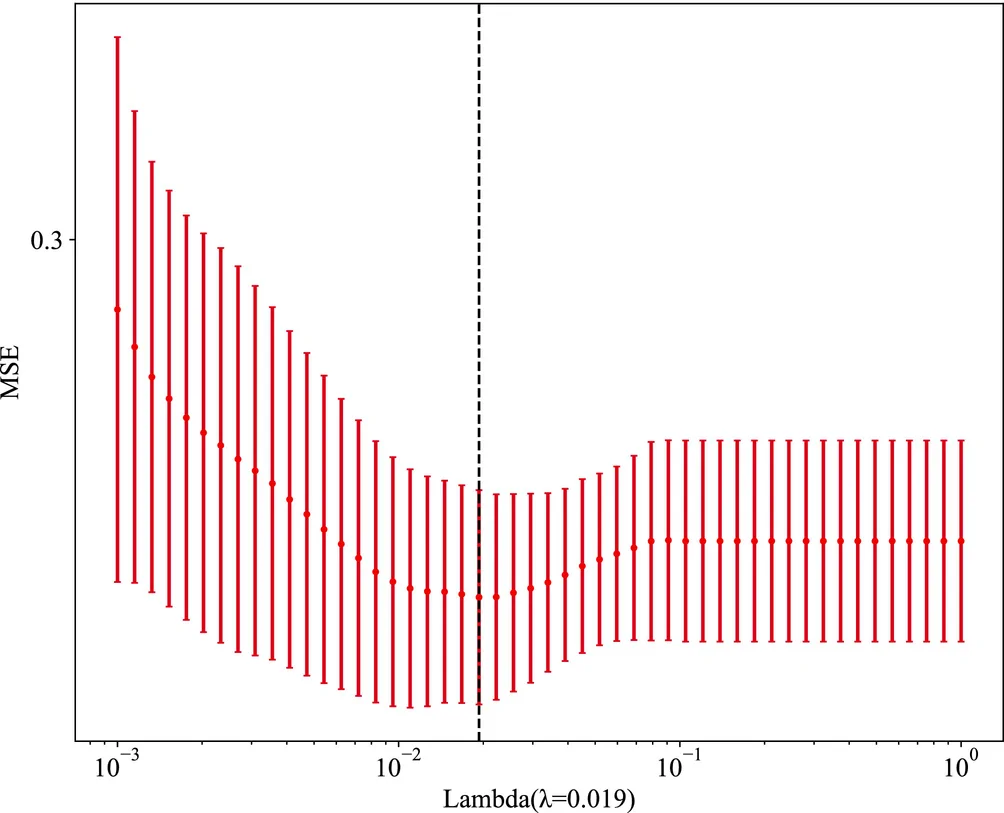
The mean squared error (MSE) of the 10‐fold cross‐validation method.

**FIGURE 7:**
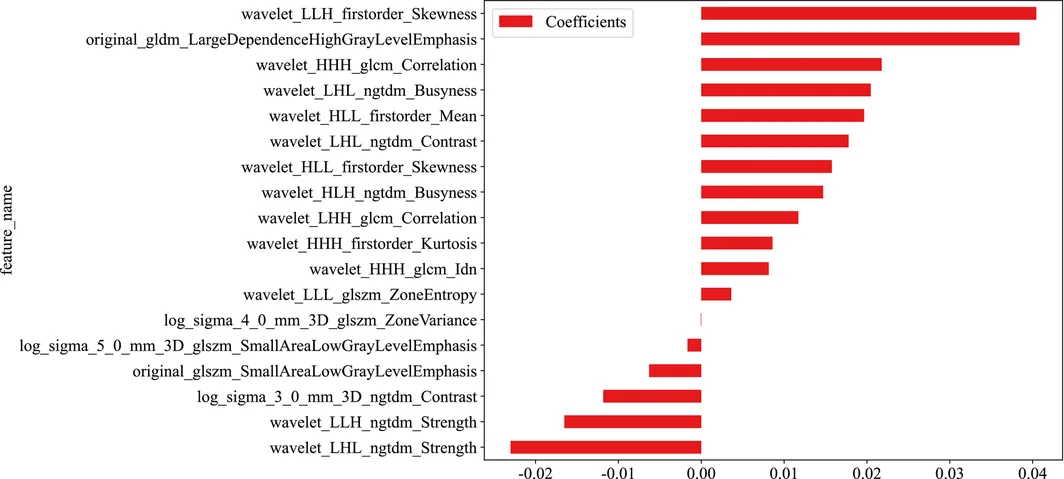
The histogram of the Rad‐score based on the selected features.

Perform radiomic feature selection on the training set: First, calculate the Pearson correlation coefficients between all features. For feature pairs with a correlation coefficient > 0.9, randomly remove one to eliminate highly redundant features. Second, perform univariate analysis (Mann–Whitney U test) on the remaining features to identify those significantly associated with LVI status (*p* < 0.05). Third, further screening was performed using univariate logistic regression, and features with a *p* value < 0.05 in the univariate logistic regression analysis were retained. Finally, the Least Absolute Shrinkage and Selection Operator (LASSO) regression combined with 10‐fold cross‐validation was employed to determine the optimal penalty coefficient *λ*, identifying the subset of nonzero coefficient features with the highest predictive value (Figures 5, 6, 7) for subsequent modeling.

#### 1.4.3 Construction and Evaluation of Imaging Informatics Models

The final selected radiomics features from the training set were used to construct models through various machine learning methods, including logistic regression (LR), support vector machine (SVM), K‐nearest neighbors (KNN), random forest (RF), extreme trees (ET), extreme gradient boosting (XGBoost), light gradient boosting machine (LightGBM), gradient boosting machine (GBM), adaptive boosting (AdaBoost), and multilayer perceptron (MLP). (LightGBM), gradient boosting machine (GBM), adaptive boosting (AdaBoost), and multilayer perceptron (MLP). Receiver operating characteristic (ROC) curves were plotted to evaluate the predictive performance of these 10 models for breast cancer LVI. Calculate corresponding sensitivity, specificity, accuracy, positive predictive value (PPV), and negative predictive value (NPV), then validate these metrics on the validation and test sets. Machine learning models were parameterized using grid search optimization. The DeLong test was applied to compare AUC differences among the 10 radiomics models, identifying the optimal radiomics model.

#### 1.4.4 Construction and Validation of Clinical and Combined Models

Using univariate and multivariate logistic regression methods, independent risk factors were identified from patients' clinical and pathological characteristics to construct clinical models. These independent risk factors were then combined with the optimal radiomics model (typically represented by its output, the radiomics score, Rad‐score) to build a combined predictive model, visualized as a column chart. The diagnostic performance of the three prediction models—the optimal radiomics model, the clinical model, and the combined model—was evaluated using the area under the ROC curve (AUC). Accuracy, sensitivity, specificity, PPV, and NPV were calculated and validated in the validation and test cohorts. Additionally, the reliability of the models is validated using the Hosmer–Lemeshow goodness‐of‐fit test. DCA is employed to assess the clinical utility of the predictive models, aiding in understanding their potential benefits within clinical settings.

### 1.5 Statistical Methods

Data analysis was performed using statistical software SPSS 26.0 and R 3.6.0 (https://www.r‐project.org). The Shapiro–Wilk test was used to assess the normality of clinical characteristics. Continuous variables meeting normality requirements were expressed as the mean ± standard deviation and compared using independent‐samples *t*‐tests. Non‐normally distributed variables were presented as the median (interquartile range) and compared using the Mann–Whitney U test. Categorical variables were expressed as case counts (percentages) and analyzed using chi‐square (*χ* ^2^) tests. Model performance was assessed using the area under the receiver operating characteristic curve (AUC), sensitivity, specificity, positive predictive value (PPV), negative predictive value (NPV), and accuracy. Model calibration was evaluated using calibration curves and the Hosmer–Lemeshow test. Clinical utility was assessed via DCA. Differences in AUC between models were compared using the DeLong test. All tests were two‐sided, and *p* < 0.05 was considered statistically significant. For radiomics feature selection, univariate logistic regression was applied with a significance level of *p* < 0.05 to identify features associated with LVI status before LASSO regression.

## 2 Results

### 2.1 Clinical and Pathological Characteristics of Patient Cohorts

There were no statistically significant differences between the training set, internal validation set, and external test set in terms of MRI‐measured tumor size, menopausal status, histological grade, sentinel lymph node status, ER, HER‐2, and Ki67 expression levels (all *p* > 0.05). However, statistically significant differences existed among the three cohorts in age, family history of breast cancer, PR status, molecular subtype, breast tissue type (lump/non‐lump), TIC type, and MRI‐assessed axillary lymph node status (*p* < 0.05). Baseline characteristics for each cohort are detailed in Table 1.

**TABLE 1:** Baseline characteristics of our cohorts.

feature_name	ALL (*n* = 912)	Train (*n* = 529)	Val (*n* = 228)	Test (*n* = 155)	*p*
Age (Mean ± SD)	50.711 ± 10.740	50.153 ± 10.537	50.215 ± 10.020	53.342 ± 12.055	0.004
MRI size (Mean ± SD)	2.761 ± 1.469	2.744 ± 1.501	2.765 ± 1.366	2.816 ± 1.510	0.864
Menopausal state					0.256
Premenopausal	387 (42.434)	213 (40.265)	101 (44.298)	73 (47.097)	
Postmenopausal	525 (57.566)	316 (59.735)	127 (55.702)	82 (52.903)	
Family history of breast cancer, *n* (%)					0.026
No	840 (92.105)	481 (90.926)	208 (91.228)	151 (97.419)	
Yes	72 (7.895)	48 (9.074)	20 (8.772)	4 (2.581)	
Histological grading, *n* (%)					0.142
1	100 (10.965)	60 (11.342)	30 (13.158)	10 (6.452)	
2	553 (60.636)	326 (61.626)	136 (59.649)	91 (58.710)	
3	259 (28.399)	143 (27.032)	62 (27.193)	54 (34.839)	
Sentinel lymph node, *n* (%)					0.519
Positive	365 (40.022)	210 (39.698)	87 (38.158)	68 (43.871)	
Negative	547 (59.978)	319 (60.302)	141 (61.842)	87 (56.129)	
ER, *n* (%)					0.096
Positive	686 (75.219)	405 (76.560)	175 (76.754)	106 (68.387)	
Negative	226 (24.781)	124 (23.440)	53 (23.246)	49 (31.613)	
PR, *n* (%)					0.023
Positive	599 (65.680)	363 (68.620)	148 (64.912)	88 (56.774)	
Negative	313 (34.320)	166 (31.380)	80 (35.088)	67 (43.226)	
HER‐2, *n* (%)					0.231
Positive	242 (26.535)	150 (28.355)	51 (22.368)	41 (26.452)	
Negative	670 (73.465)	379 (71.645)	177 (77.632)	114 (73.548)	
Ki67, *n* (%)					0.090
High expression	710 (77.851)	405 (76.560)	174 (76.316)	131 (84.516)	
Low expression	202 (22.149)	124 (23.440)	54 (23.684)	24 (15.484)	
Molecular typing, *n* (%)					< 0.001
Luminal A	148 (16.228)	91 (17.202)	37 (16.228)	20 (12.903)	
Luminal B	529 (58.004)	321 (60.681)	140 (61.404)	68 (43.871)	
HER‐2 overexpression	121 (13.268)	60 (11.342)	19 (8.333)	42 (27.097)	
Triple‐negative	114 (12.500)	57 (10.775)	32 (14.035)	25 (16.129)	
Types of mammary gland tissue, *n* (%)					< 0.001
Lump	741 (81.250)	458 (86.578)	201 (88.158)	82 (52.903)	
Non‐mass enhancement	171 (18.750)	71 (13.422)	27 (11.842)	73 (47.097)	
TIC, *n* (%)					< 0.001
I	90 (9.868)	48 (9.074)	33 (14.474)	9 (5.806)	
II	436 (47.807)	226 (42.722)	81 (35.526)	129 (83.226)	
III	386 (42.325)	255 (48.204)	114 (50.000)	17 (10.968)	
MRI of axillary lymph nodes, *n* (%)					0.028
Positive	499 (54.715)	303 (57.278)	126 (55.263)	70 (45.161)	
Negative	413 (45.285)	226 (42.722)	102 (44.737)	85 (54.839)	

Abbreviations: ER, estrogen receptor; HER‐2, human epidermal growth factor receptor 2; MRI, magnetic resonance imaging; PR, progesterone receptor; TIC, time‐signal intensity curve.

### 2.2 Screening of Radiomic Features and Model Construction

Based on DCE‐MRI images, 1197 radiomic features were extracted from each patient. A stepwise screening process was conducted on the training set: First, features with Pearson correlation coefficients below 0.9 were filtered out to eliminate highly redundant features, leaving 651 features. Next, univariate analysis retained 124 features with *p* < 0.05. Subsequently, univariate logistic regression was applied. Finally, 10‐fold cross‐validation combined with the least absolute shrinkage and selection operator (LASSO) was used to determine the optimal hyperparameter *λ* value, ultimately identifying 18 key radiomics features. Based on these features, 10 machine learning‐based radiomics models were constructed, including LR, SVM, and KNN.

### 2.3 Evaluation of Imaging Informatics Models

**FIGURE 8:**
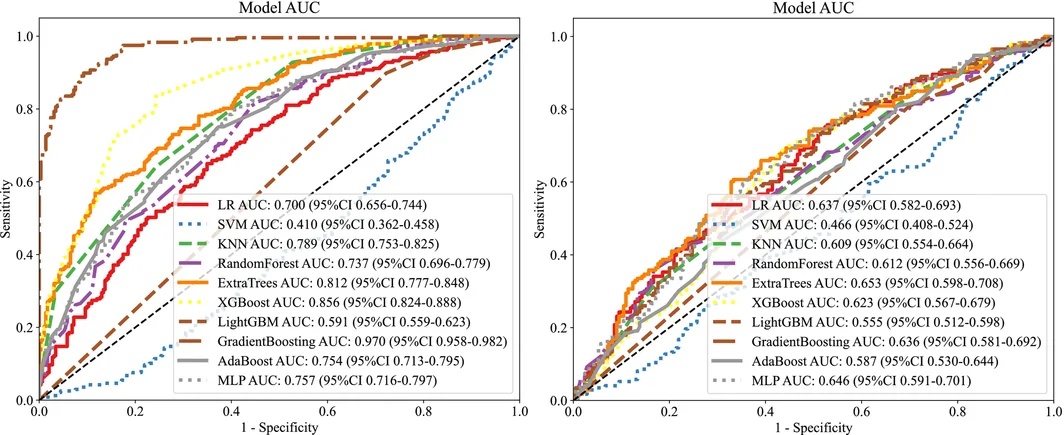
displays the receiver operating characteristic curves for 10 machine learning models predicting vascular invasion in breast cancer patients within the training and validation sets.

Based on a study utilizing MRI radiomics features, the detailed performance metrics of 10 radiomics models in predicting breast cancer LVI were evaluated using ROC curves across the training and internal validation sets. Specific results are presented in Table 2, with the ROC curves shown in Figure 8. Comprehensive evaluation revealed that the ExtraTrees model achieved the highest AUC (0.653) and accuracy (0.640) in the validation set, indicating strong generalization capability and diagnostic performance. Consequently, it was selected as the optimal radiomics model for subsequent analysis.

**TABLE 2:** Predictive performance of 10 radiomics models for lymphovascular invasion in breast cancer patients from the training and validation sets.

model_name	Task	Accuracy	AUC	95% CI	Sensitivity	Specificity	PPV	NPV
LR	train	0.641	0.700	0.6557–0.7440	0.743	0.558	0.577	0.728
LR	test	0.601	0.637	0.5819–0.6930	0.757	0.471	0.541	0.702
SVM	train	0.456	0.410	0.3617–0.4582	1.000	0.014	0.451	1.000
SVM	test	0.467	0.466	0.4077–0.5236	0.988	0.038	0.458	0.800
KNN	train	0.677	0.789	0.7527–0.8246	0.928	0.473	0.588	0.890
KNN	test	0.598	0.609	0.5540–0.6637	0.306	0.838	0.609	0.595
RandomForest	train	0.671	0.737	0.6955–0.7786	0.806	0.562	0.599	0.781
RandomForest	test	0.619	0.612	0.5557–0.6691	0.416	0.786	0.615	0.620
ExtraTrees	train	0.743	0.812	0.7768–0.8480	0.570	0.884	0.799	0.717
ExtraTrees	test	0.640	0.653	0.5977–0.7081	0.607	0.667	0.600	0.673
XGBoost	train	0.794	0.856	0.8241–0.8875	0.840	0.757	0.737	0.853
XGBoost	test	0.621	0.623	0.5672–0.6794	0.694	0.562	0.566	0.690
LightGBM	train	0.556	0.591	0.5590–0.6231	0.899	0.277	0.502	0.771
LightGBM	test	0.530	0.555	0.5115–0.5979	0.809	0.300	0.488	0.656
GradientBoosting	train	0.904	0.970	0.9579–0.9822	0.899	0.908	0.887	0.917
GradientBoosting	test	0.619	0.636	0.5811–0.6919	0.549	0.676	0.583	0.645
AdaBoost	train	0.682	0.754	0.7134–0.7947	0.751	0.627	0.620	0.756
AdaBoost	test	0.546	0.587	0.5303–0.6441	0.809	0.329	0.498	0.676
MLP	train	0.688	0.757	0.7164–0.7974	0.797	0.599	0.618	0.785
MLP	test	0.624	0.646	0.5913–0.7014	0.607	0.638	0.580	0.663

Abbreviations: AUC, area under the receiver operating characteristic curve; NPV, negative predictive value; PPV, positive predictive value.

### 2.4 Construction and Evaluation of Clinical Predictive Models

Univariate and multivariate logistic regression analyses revealed that sentinel lymph node status, ER, and TIC were independent risk factors for predicting breast cancer LVI (see Table 3 for details). Based on these three independent risk factors, we constructed a clinical prediction model.

**TABLE 3:** Univariable analysis of clinical features.

feature_name	Single‐factor analysis		Multivariate analysis	
	OR (95% CI)	*p*	OR (95% CI)	*p*
Age	0.995 (0.992–0.998)	0.003	1.004 (0.990–1.019)	0.626
Menopausal state	0.926 (0.850–1.009)	0.138		
Histological grading	0.945 (0.886–1.007)	0.146		
Sentinel lymph node	0.755 (0.691–0.824)	0.000	0.171 (0.121–0.243)	0.000
ER	0.865 (0.775–0.966)	0.031	2.396 (1.359–4.221)	0.011
PR	0.850 (0.766–0.943)	0.010	0.826 (0.486–1.405)	0.554
HER‐2	0.871 (0.803–0.946)	0.005	1.495 (1.064–2.100)	0.052
Ki67	0.790 (0.706–0.883)	0.001	0.664 (0.449–0.981)	0.085
Molecular typing	0.929 (0.874–0.989)	0.053		
TIC	0.915 (0.863–0.970)	0.012	1.355 (1.080–1.701)	0.028
Types of mammary gland tissue	0.827 (0.732–0.934)	0.010	1.566 (0.974–2.517)	0.120
MRI of axillary lymph nodes	0.811 (0.736–0.893)	0.000	1.046 (0.757–1.445)	0.821
MRI size	1.000 (0.955–1.047)	0.998		
Family history of breast cancer	1.087 (0.676–1.749)	0.773		

Abbreviations: ER, estrogen receptor; HER‐2, human epidermal growth factor receptor‐2; MRI, magnetic resonance imaging; PR, progesterone receptor; TIC, time signal intensity curve.

### 2.5 Construction, Evaluation, and Visualization of Combined Models

**FIGURE 9:**
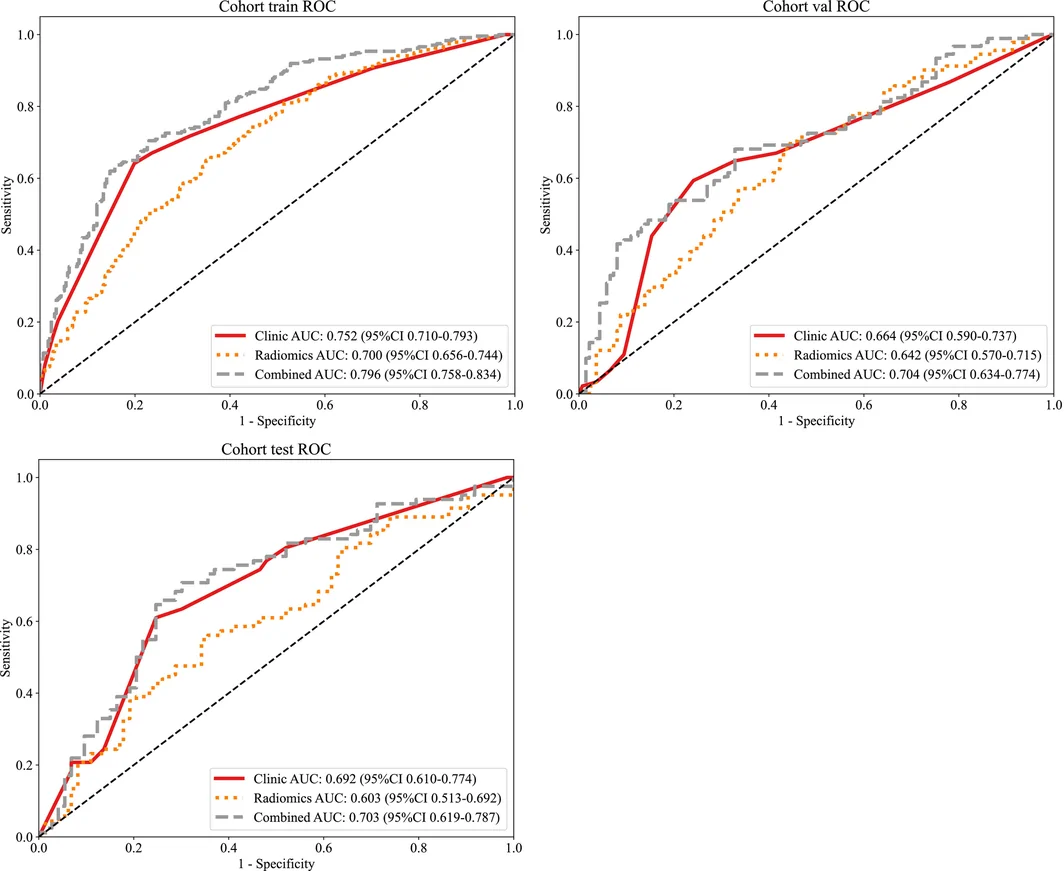
Different signatures ROC on different cohorts.

**FIGURE 10:**
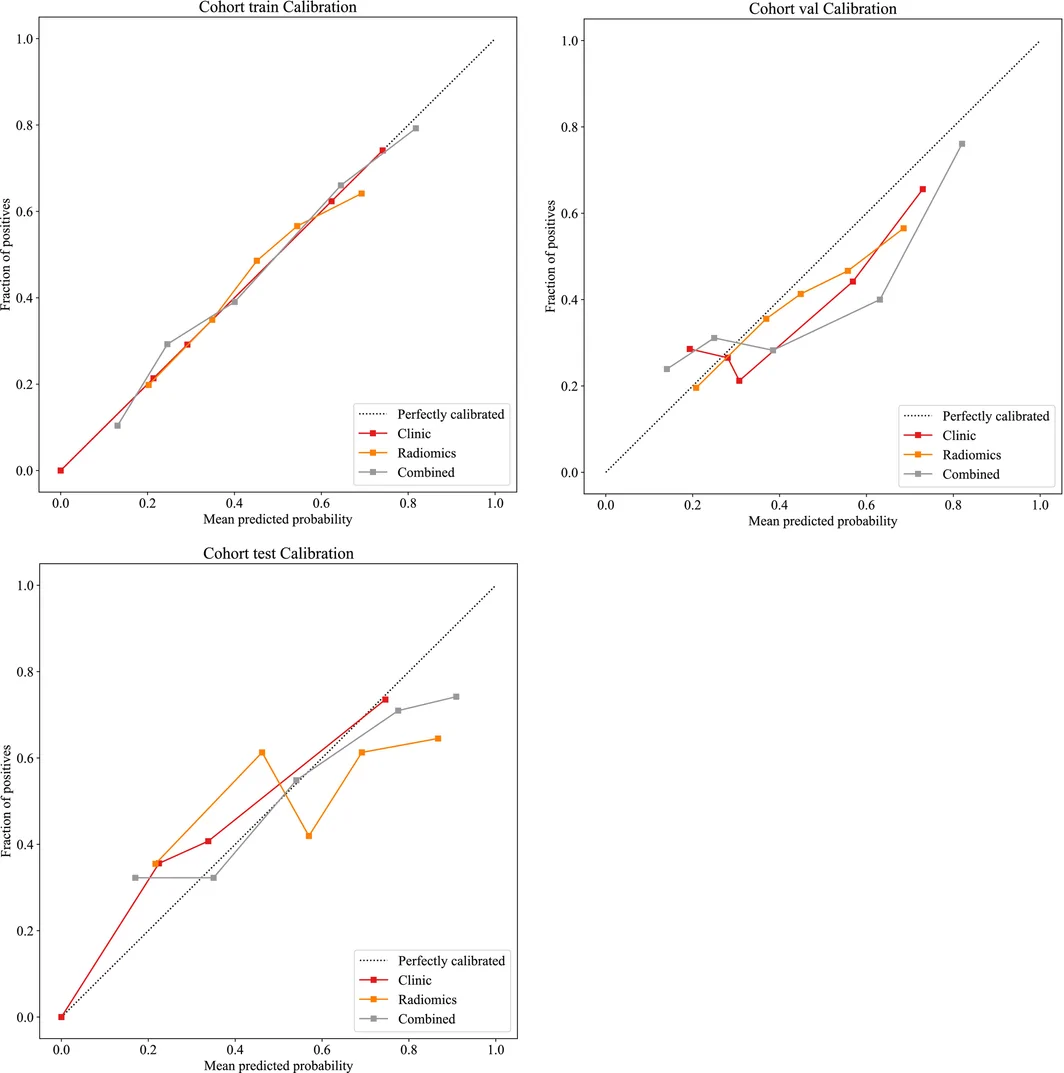
Calibration curves of different signatures in the cohort.

**FIGURE 11:**
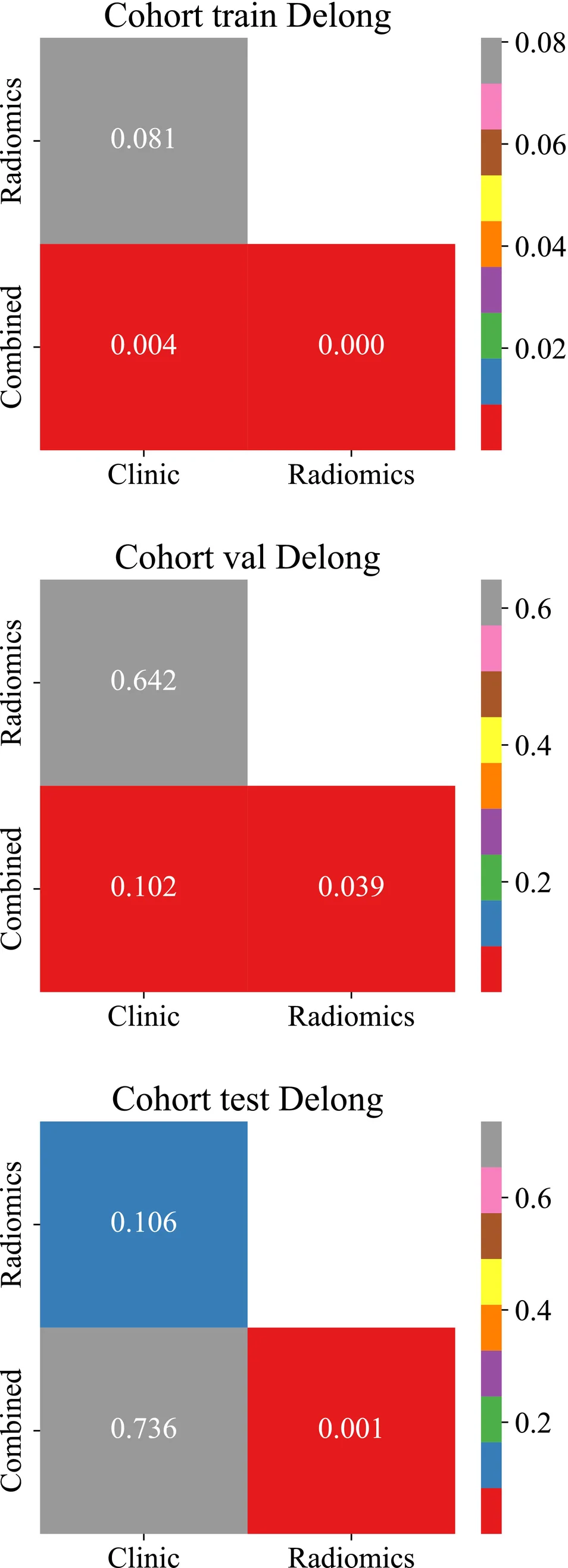
Results of DeLong for different signatures.

**FIGURE 12:**
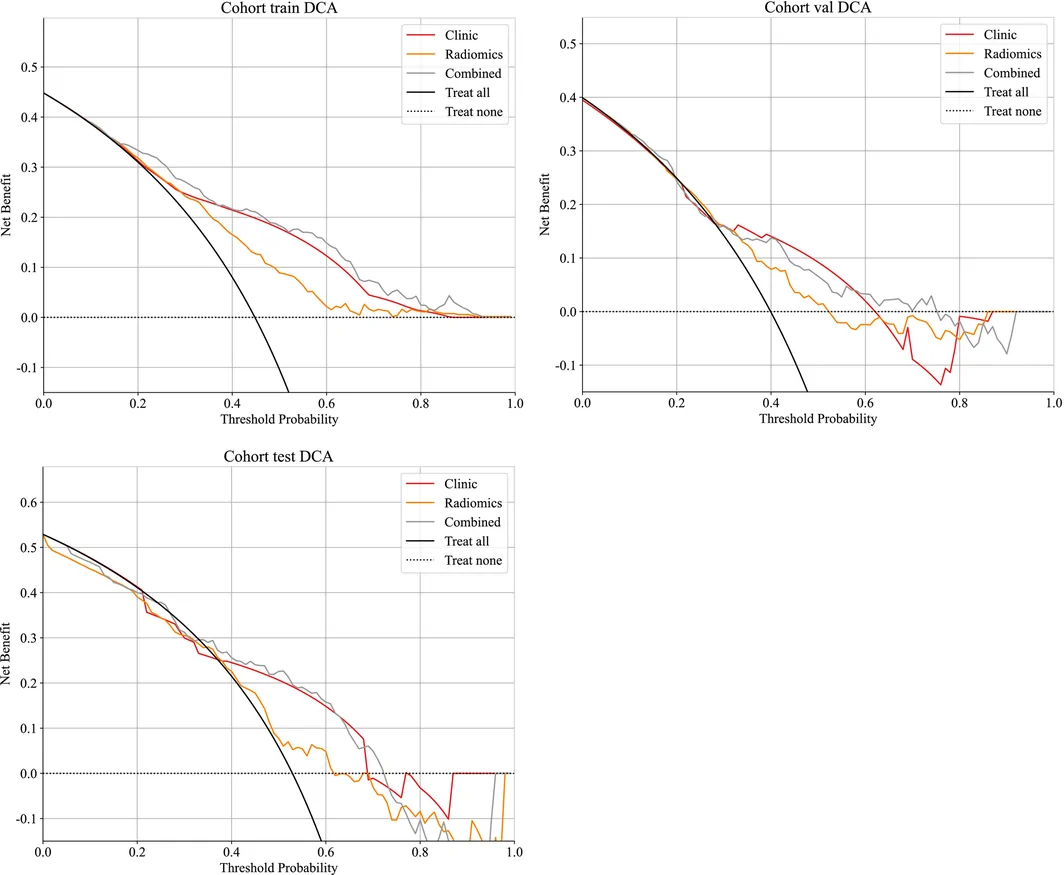
Decision curves of different signatures in the cohort.

**FIGURE 13:**
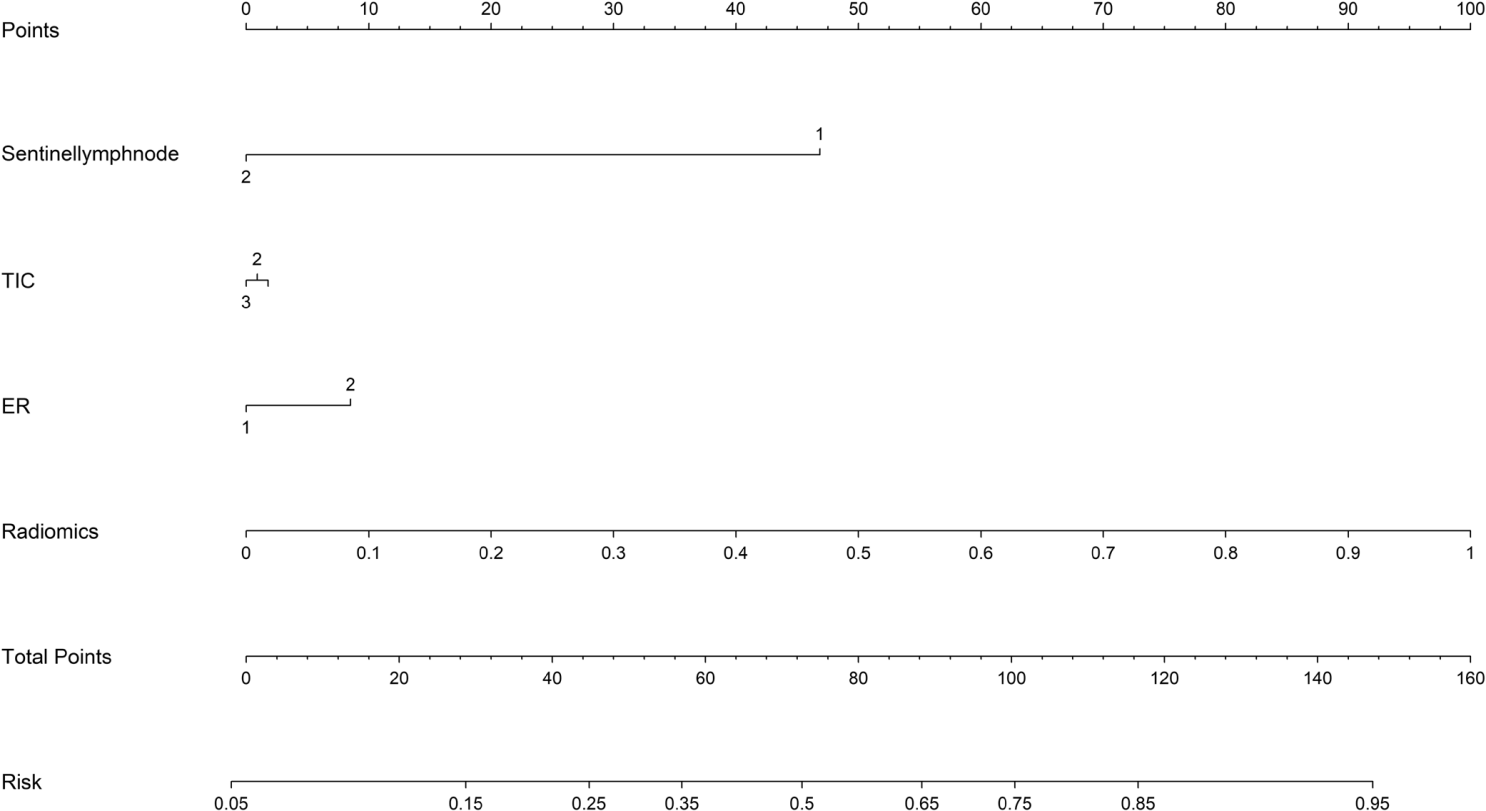
A predictive scorecard integrating clinical and pathological factors, as demonstrated in the study of ER‐positive breast cancer patients.

By integrating the best‐performing radiomics model (ExtraTrees) with clinically independent risk factors, a combined predictive model was constructed. The performance of the radiomics model, clinical model, and combined model was evaluated using AUC values. On the training set, the radiomics model achieved an AUC of 0.700 (95% CI: 0.6557–0.7440), the clinical model's AUC was 0.752 (95% CI: 0.7101–0.7933), while the combined model's AUC reached 0.796 (95% CI: 0.7582–0.8341). On the validation set, the corresponding AUC values were 0.642 (95% CI: 0.5695–0.7145), 0.664 (95% CI: 0.5901–0.7374), and 0.704 (95% CI: 0.6339–0.7745). Finally, on the test set, the AUC values for the radiomics model, clinical model, and combined model were 0.603 (95% CI: 0.5131–0.6923), 0.692 (95% CI: 0.6097–0.7739), and 0.703 (95% CI: 0.6187–0.7866). These results indicate that the combined model demonstrates high predictive performance across different datasets. As shown in Figure 9 and Table 4, the AUC of the combined model exceeded that of either the standalone radiomics model or the clinical model across the training set, internal validation set, and external test set. Calibration curves revealed good agreement between observed and predicted outcomes for the training and validation sets, indicating favorable calibration performance (Figure 10). DeLong test: Applied across training, validation, and test sets to assess the statistical significance of model differences, the combined model demonstrated significant improvement over both radiomics and clinical models (Figure 11). DCA: Figure 12 presents DCA results for the training, validation, and test sets. Findings indicate our combined model holds a significant advantage in predictive probability. Furthermore, compared to other features, this model consistently demonstrates greater potential net benefit, suggesting both the combined model and its columnar graph possess strong clinical utility. Construction of the Predictive Nomogram: To further enable personalized prediction, we integrated clinically‐imaging features with independent predictive value from multifactorial analysis alongside the radiological score (Rad‐score) to construct a visual predictive nomogram (Figure 13). This nomogram facilitates intuitive estimation of the probability of LVI occurrence in breast cancer patients.

**TABLE 4:** Metrics on different signatures.

Signature	Accuracy	AUC	95% CI	Sensitivity	Specificity	PPV	NPV	Cohort
Clinic	0.730	0.752	0.7101–0.7933	0.641	0.801	0.724	0.734	train
Radiomics	0.641	0.700	0.6557–0.7440	0.743	0.558	0.577	0.728	train
Combined	0.749	0.796	0.7582–0.8341	0.637	0.839	0.763	0.740	train
Clinic	0.693	0.664	0.5901–0.7374	0.593	0.759	0.621	0.738	val
Radiomics	0.614	0.642	0.5695–0.7145	0.703	0.555	0.512	0.738	val
Combined	0.675	0.704	0.6339–0.7745	0.681	0.672	0.579	0.760	val
Clinic	0.677	0.692	0.6097–0.7739	0.610	0.753	0.735	0.632	test
Radiomics	0.600	0.603	0.5131–0.6923	0.549	0.658	0.643	0.565	test
Combined	0.703	0.703	0.6187–0.7866	0.707	0.699	0.725	0.680	test

Abbreviations: AUC, area under the receiver operating characteristic curve; NPV, negative predictive value; PPV, positive predictive value.

## 3 Discussion

This study developed and validated a combined model for preoperative prediction of LVI based on dynamic contrast‐enhanced MRI (DCE‐MRI) data from 912 breast cancer patients across two independent medical centers. Our core finding is that the combined model, integrating radiomic features with key clinical semantic features (sentinel lymph node status, ER status, and TIC type), consistently outperformed either standalone radiomic or clinical models in predictive performance (measured by AUC) across both the internal validation set and an independent external test set. Receiver operating characteristic curve analysis further confirmed that this combined model demonstrated higher clinical net benefit across a broad range of threshold probabilities. These results suggest that DCE‐MRI‐based combined models offer a promising adjunctive tool for preoperative, noninvasive, and individualized assessment of breast cancer LVI risk, thereby aiding in optimized treatment decision‐making.

The design and execution of this study offer several significant advantages. First, we adopted a rigorous multi‐center external validation strategy. Unlike most previous single‐center studies or those relying solely on internal cross‐validation [14, 15, 16], we utilized data from 155 patients at another hospital as an independent test set. This substantially enhances the reliability of model generalization assessment and its clinical translation potential. Second, we systematically compared 10 mainstream machine learning algorithms. Unlike studies employing fewer than three algorithms (e.g., logistic regression or support vector machines) [15, 17, 18], our systematic screening ensured the selected “best” model (Extreme Random Trees, ExtraTrees) resulted from thorough comparison, minimizing the risk of overlooking superior solutions due to algorithm selection bias. Finally, our combined model does not merely combine radiomics and clinical features into a single model. Instead, it first identifies independent clinical predictors through multivariate analysis and then integrates them with the outputs from the best‐performing radiomics model. This strategy, validated in studies by Jiang et al. [19] and Pan et al. [20], effectively avoids the dimensionality disaster and overfitting issues in the feature space while fully leveraging the complementary advantages of different modalities.

However, comparing the results of this study with existing literature reveals several noteworthy differences and challenges. Multiple earlier studies reported high predictive performance for LVI using MRI radiomics, with AUC values exceeding 0.80 [21, 22, 23]. In contrast, the best radiomics model in this study achieved an AUC of 0.653 on the external validation set, while the combined model reached 0.703—relatively conservative values. This discrepancy may stem from several factors: first, differences in study populations and inclusion criteria. This study encompassed all molecular subtypes of breast cancer, including Luminal A, Luminal B, HER‐2 overexpressing, and triple‐negative types, with non‐mass‐type enhancing lesions accounting for 18.75% of the cohort. Some literature may focus on specific subtypes or nodular lesions, exhibiting higher case homogeneity that may facilitate model learning of specific patterns. The clinical‐pathological characteristics of the patient cohort in this study reveal significant differences in molecular typing, lesion type, and TIC distribution between the external test set and development set across multicenter clinical trials, authentically reflecting data heterogeneity. This heterogeneity objectively contributes to model performance “degradation” in certain scenarios, yet simultaneously demonstrates the model's robustness against real‐world data variability. As demonstrated by Ghulam B et al. [24], sources of molecular testing data heterogeneity include sample types, detection technologies, and biological variations—factors particularly pronounced in multicenter studies. Furthermore, Zhao et al.'s [25] multicenter breast cancer MRI analysis indicated that peritumoral and intertumoral heterogeneity significantly impacts predicting breast cancer LVI, yielding AUC values of 0.951 (training) and 0.929 (validation) with high net gain in DCA. This further underscores the importance of accounting for heterogeneity in multicenter studies. Second, differences in research methodology and validation rigor. Many studies reporting high AUC values employed single‐center data or lenient validation approaches (e.g., training‐test splitting or cross‐validation alone) [26], failing to adequately assess model robustness across scanning protocols, equipment, and population variations. While our rigorous external validation resulted in “lower” performance metrics, it provides a more realistic and stringent assessment of the model's practical application potential. Third, differences in feature engineering and model construction. We employed a multistep feature reduction strategy involving correlation filtering, univariate screening, and LASSO regression, ultimately selecting 18 features from 1197. While this approach enhances model stability and interpretability, it may sacrifice some weak signals with combined predictive value. Some studies may have utilized more complex deep learning or alternative feature selection methods, potentially explaining technical differences in performance [27, 28].

Although the combined model in this study demonstrated consistent advantages, we must acknowledge its limitations. First, the inherent selection bias of retrospective study designs cannot be entirely avoided. Second, despite the large overall sample size, the sample size of the independent validation cohort (*n* = 155) remains relatively limited, particularly considering the distribution of LVI‐positive versus LVI‐negative cases. This may impact the precision of performance evaluation. Future validation requires prospective, multicenter studies with larger samples. Third, tumor delineation currently relies on manual operation by experienced radiologists. Although quality is ensured through dual‐reader review and arbitration, this process remains time‐consuming and susceptible to inter‐observer variability. Future exploration of robust semi‐automated or fully automated segmentation algorithms will facilitate standardization and broader adoption of this technology [29, 30, 31]. Fourth, the model developed in this study has not been directly compared with the Breast Imaging Reporting and Data System (BI‐RADS) lexicon or other clinical prediction rules, and its incremental value within existing clinical workflows requires further clarification. Fifth, regarding inter‐center acquisition variability, both participating centers used the same MRI scanner (Philips Ingenia 3.0T) and vendor, which minimized hardware‐related heterogeneity. However, minor differences in sequence parameters between the two centers may still affect radiomic feature reproducibility. Importantly, no dedicated image harmonization strategy (e.g., ComBat) was applied prior to feature extraction. This lack of harmonization could have contributed to the observed performance drop in the external test set and represents a limitation of the current study. Future multicenter radiomics studies should consider incorporating such harmonization techniques to improve model generalizability.

In summary, this study developed and rigorously validated a combined model based on multicenter DCE‐MRI, which can reliably predict breast cancer LVI status preoperatively with good calibration and clinical utility. Future research directions should include: conducting prospective validation studies to confirm its clinical efficacy; integrating multi‐parametric MRI information (e.g., DWI, ADC values) to enrich the feature system, particularly for breast cancer LVI prediction; exploring explainable AI (XAI) techniques to reveal the imaging basis of model decisions; and ultimately attempting to integrate it into clinical decision support systems to evaluate its practical impact on treatment decisions and patient prognosis.

## Author Contributions

**Hongen Li:** conceptualization, methodology, software, writing – original draft, data curation. **Qingwen Xiao:** investigation, validation, formal analysis, supervision. **Yihui Zeng:** funding acquisition, visualization. **Xia Wang:** project administration, resources. **Yan Zhang:** writing – original draft, writing – review and editing.

## Funding

The authors have nothing to report.

## Disclosure

No artificial intelligence tools were used in the preparation of this manuscript.

## Ethics Statement

This study is a retrospective, multicenter investigation conducted in accordance with the Declaration of Helsinki. The research protocol was approved by the institutional medical ethics committees of both participating centers (Center 1: Guangdong Maternal and Child Health Hospital, Approval No. 20261015; Center 2: The First Affiliated Hospital of Jinan University, Approval No. KY‐2025‐180), and patients were exempted from providing informed consent.

## Consent

The authors have nothing to report.

## Conflicts of Interest

The authors declare no conflicts of interest.

## Data Availability

The data that support the findings of this study were obtained from two medical centers (Guangdong Women and Children Hospital and The First Affiliated Hospital of Jinan University) and are subject to ethical and privacy restrictions, as the study involves sensitive patient‐level clinical and imaging data. Therefore, the datasets are not publicly available. However, de‐identified individual patient‐level data may be made available from the corresponding author upon reasonable request, subject to approval by the institutional ethics committees of both participating centers and the signing of a data access agreement. Requests for data access should be submitted in writing to the corresponding author (Yan Zhang, doctorzhangyan@vip.163.com), clearly specifying the intended use and scope of the requested data. Responses to data requests will be provided within 4 weeks of submission. All shared data will be fully anonymized to protect patient privacy, in compliance with the Declaration of Helsinki and applicable data protection regulations.

## References

[1] R. L. Siegel , A. N. Giaquinto , and A. Jemal , “Cancer Statistics, 2024,” CA: A Cancer Journal for Clinicians 74, no. 1 (2024): 12–49. 38230766 10.3322/caac.21820

[2] J. Kim , A. Harper , V. McCormack , et al., “Global Patterns and Trends in Breast Cancer Incidence and Mortality Across 185 Countries,” Nature Medicine 31, no. 4 (2025): 1154–1162. 10.1038/s41591-025-03502-3 39994475

[3] C. Shen , C. Lin , F. Qu , et al., “Genomic Spectra of Lymphovascular Invasion in Breast Cancer,” Chinese Journal of Cancer Research = Chung‐Kuo Yen Cheng Yen Chiu 37, no. 2 (2025): 138–153. 40353081 10.21147/j.issn.1000-9604.2025.02.02 PMC12062984

[4] H. Wang , C. Huang , Y. Wang , et al., “Preoperative Imaging Assessment of Lymphovascular Invasion in Breast Cancer: Current Evidence, Technical Advances, and Future Directions,” Breast Cancer (Dove Medical Press) 17 (2025): 1051–1062. 41245007 10.2147/BCTT.S562537 PMC12619624

[5] C. Shen , C. Lin , F. Qu , et al., “Genomic Spectra of Lymphovascular Invasion in Breast Cancer,” Chinese Journal of Cancer Research 37, no. 2 (2025): 138–153. 40353081 10.21147/j.issn.1000-9604.2025.02.02 PMC12062984

[6] H. Shen , T. Zhang , and S. Wang , “A Prediction Model for Lymph Node Metastasis of Oral Squamous Cell Carcinoma Based on Multiple Risk Factors,” Cllinical and Experimental Dental Research 10, no. 6 (2024): e70046. 10.1002/cre2.70046 PMC11570548 39552015

[7] Y. Yang , H. Wei , F. Fu , et al., “Preoperative Prediction of Lymphovascular Invasion of Colorectal Cancer by Radiomics Based on 18F‐FDG PET‐CT and Clinical Factors,” Frontiers in Radiology 3 (2023): 1212382. 37614530 10.3389/fradi.2023.1212382 PMC10442652

[8] Z. Wang , F. Li , J. Cai , et al., “Identification of Lesion Bioactivity in Hepatic Cystic Echinococcosis Using a Transformer‐Based Fusion Model,” Journal of Infection 90, no. 4 (2025): 106455. 40049526 10.1016/j.jinf.2025.106455

[9] L. Russo , D. Charles‐Davies , S. Bottazzi , E. Sala , and L. Boldrini , “Radiomics for Clinical Decision Support in Radiation Oncology,” Clinical Oncology (Royal College of Radiologists) 36, no. 8 (2024): e269–e281. 10.1016/j.clon.2024.03.003 38548581

[10] G. Liang , S. Zhang , Y. Zheng , et al., “Establishment of a Predictive Nomogram for Breast Cancer Lympho‐Vascular Invasion Based on Radiomics Obtained From Digital Breast Tomography and Clinical Imaging Features,” BMC Medical Imaging 25, no. 1 (2025): 65. 40011817 10.1186/s12880-025-01607-2 PMC11866887

[11] M. Xu , H. Yang , J. Sun , H. Hao , X. Li , and G. Liu , “Development of an Intratumoral and Peritumoral Radiomics Nomogram Using Digital Breast Tomosynthesis for Preoperative Assessment of Lymphovascular Invasion in Invasive Breast Cancer,” Academic Radiology 31, no. 5 (2024): 1748–1761. 38097466 10.1016/j.acra.2023.11.010

[12] C. J. Robbins , K. M. Bates , and D. L. Rimm , “HER2 Testing: Evolution and Update for a Companion Diagnostic Assay,” Nature Reviews. Clinical Oncology 22, no. 6 (2025): 408–423. 10.1038/s41571-025-01016-y PMC12903097 40195456

[13] A. Goldhirsch , E. P. Winer , A. S. Coates , et al., “Personalizing the Treatment of Women With Early Breast Cancer: Highlights of the St Gallen International Expert Consensus on the Primary Therapy of Early Breast Cancer 2013,” Annals of Oncology 24, no. 9 (2013): 2206–2223. 23917950 10.1093/annonc/mdt303 PMC3755334

[14] C. Zhang , P. Zhou , R. Li , et al., “Prediction of Lymphovascular Invasion in Invasive Breast Cancer Based on Clinical‐MRI Radiomics Features,” BMC Medical Imaging 24, no. 1 (2024): 277. 39415127 10.1186/s12880-024-01456-5 PMC11481431

[15] X. Li , K. Luo , N. Zhang , et al., “Prediction of Lymphovascular Invasion Status in Breast Cancer Based on Magnetic Resonance Imaging Radiomics Features,” Magnetic Resonance Imaging 109 (2024): 91–95. 38467265 10.1016/j.mri.2024.03.008

[16] Q. Ma , X. Lu , Q. Chen , H. Gong , and J. Lei , “Multiphases DCE‐MRI Radiomics Nomogram for Preoperative Prediction of Lymphovascular Invasion in Invasive Breast Cancer,” Academic Radiology 31, no. 12 (2024): 4743–4758. 39107190 10.1016/j.acra.2024.06.007

[17] W. Liu , L. Li , J. Deng , and W. Li , “A Comprehensive Approach for Evaluating Lymphovascular Invasion in Invasive Breast Cancer: Leveraging Multimodal MRI Findings, Radiomics, and Deep Learning Analysis of Intra‐ and Peritumoral Regions,” Computerized Medical Imaging and Graphics 116 (2024): 102415. 39032451 10.1016/j.compmedimag.2024.102415

[18] H. Zheng , L. Jian , L. Li , W. Liu , and W. Chen , “Delta‐Radiomics Based on Dynamic Contrast‐Enhanced MRI for Predicting Lymphovascular Invasion in Invasive Breast Cancer,” Academic Radiology 31, no. 5 (2024): 1762–1772. 38092588 10.1016/j.acra.2023.11.017

[19] Y. Jiang , Y. Zeng , Z. Zuo , et al., “Leveraging Multimodal MRI‐Based Radiomics Analysis With Diverse Machine Learning Models to Evaluate Lymphovascular Invasion in Clinically Node‐Negative Breast Cancer,” Heliyon 10, no. 1 (2023): e23916. 38192872 10.1016/j.heliyon.2023.e23916 PMC10772250

[20] G. Pan , Z. Pan , W. Chen , et al., “Integration of MRI Radiomics and Clinical Data for Preoperative Prediction of Vascular Invasion in Breast Cancer: A Deep Learning Approach,” Magnetic Resonance Imaging 118 (2025): 110339. 39880177 10.1016/j.mri.2025.110339

[21] R. Liang , F. Li , J. Yao , et al., “Predictive Value of MRI‐Based Deep Learning Model for Lymphovascular Invasion Status in Node‐Negative Invasive Breast Cancer,” Scientific Reports 14, no. 1 (2024): 16204. 39003325 10.1038/s41598-024-67217-0 PMC11246470

[22] S. Chen , Z. Zhong , Y. Chen , et al., “Prediction of Lymphovascular Invasion in Invasive Breast Cancer via Intratumoral and Peritumoral Multiparametric Magnetic Resonance Imaging Machine Learning‐Based Radiomics With Shapley Additive Explanations Interpretability Analysis,” Quantitative Imaging in Medicine and Surgery 15, no. 9 (2025): 7833–7846. 40893558 10.21037/qims-2024-2685 PMC12397653

[23] F. Han , W. Li , Y. Hu , H. Wang , T. Liu , and J. Wu , “MRI Radiomics‐Based Machine Learning to Predict Lymphovascular Invasion of HER2‐Positive Breast Cancer,” Journal of Imaging Informatics in Medicine 38, no. 4 (2025): 2012–2020. 39538052 10.1007/s10278-024-01329-x PMC12343420

[24] B. Ghulam , Z. Bashir , A. K. Akram , et al., “C‐Reactive Protein (CRP) in Patients With Myocarditis: A Systematic Review and Meta‐Analysis,” Cureus 16, no. 10 (2024): e71885. 39564010 10.7759/cureus.71885 PMC11573699

[25] Q. Zhao , H. Zhang , and W. Xing , “Integrating Peritumoral and Intratumoral Radiomics With Deep Learning for Preoperative Prediction of Lymphovascular Invasion in Invasive Breast Cancer Using DCE‐MRI,” Technology in Cancer Research and Treatment 24 (2025): 15330338251374945. 40899931 10.1177/15330338251374945 PMC12409024

[26] H. Zheng , L. Jian , L. Li , W. Liu , and W. Chen , “Prior Clinico‐Radiological Features Informed Multi‐Modal MR Images Convolution Neural Network: A Novel Deep Learning Framework for Prediction of Lymphovascular Invasion in Breast Cancer,” Cancer Medicine 13, no. 3 (2024): e6932. 38230837 10.1002/cam4.6932 PMC10905682

[27] Y. He , Z. Zuo , Y. Li , X. Yang , Y. Feng , and L. He , “Prior‐Radiomics‐Guided Multi‐Scale Feature Extraction Network Utilizing Preoperative MRI: A Pioneering Approach for Lymphovascular Invasion Prediction in Invasive Breast Cancer,” Technology in Cancer Research and Treatment 24 (2025): 15330338251391862. 41212687 10.1177/15330338251391862 PMC12602961

[28] J. Xu , G. Wang , Y. Wei , et al., “Multi‐Parameter MRI Deep Learning Model for Lymphovascular Invasion Assessment in Invasive Breast Ductal Carcinoma: A Multicenter, Retrospective Study,” Clinical Radiology 88 (2025): 107002. 40695008 10.1016/j.crad.2025.107002

[29] G. Sanabria‐Diaz , A. Cagol , P. J. Lu , et al., “Advanced MRI Measures of Myelin and Axon Volume Identify Repair in Multiple Sclerosis,” Annals of Neurology 97, no. 1 (2024): 134–148. 39390658 10.1002/ana.27102 PMC11683175

[30] L. Coll , D. Pareto , F. Aparicio‐Serrano , et al., “Deep Learning to Predict Independent Progression of Relapse Activity at a First Demyelinating Event,” Brain Commun 7, no. 4 (2025): FCAF243. 40620473 10.1093/braincomms/fcaf243 PMC12226453

[31] J. Yin , L. Liu , X. Luo , et al., “MHAU‐Net: A Multi‐Scale Hybrid Attention U‐Shaped Network for the Segmentation of MRI Breast Tumors,” Quantitative Imaging in Medicine and Surgery 15, no. 5 (2025): 4758–4773. 40384695 10.21037/qims-24-1515 PMC12082575

